# Shape-shifting colloids via stimulated dewetting

**DOI:** 10.1038/ncomms12216

**Published:** 2016-07-18

**Authors:** Mena Youssef, Theodore Hueckel, Gi-Ra Yi, Stefano Sacanna

**Affiliations:** 1 Department of Chemistry, Molecular Design Institute, New York University, 29 Washington Place, New York, New York 10003, USA; 2 School of Chemical Engineering, Sungkyunkwan University (SKKU), Suwon 440-746, South Korea

## Abstract

The ability to reconfigure elementary building blocks from one structure to another is key to many biological systems. Bringing the intrinsic adaptability of biological systems to traditional synthetic materials is currently one of the biggest scientific challenges in material engineering. Here we introduce a new design concept for the experimental realization of self-assembling systems with built-in shape-shifting elements. We demonstrate that dewetting forces between an oil phase and solid colloidal substrates can be exploited to engineer shape-shifting particles whose geometry can be changed on demand by a chemical or optical signal. We find this approach to be quite general and applicable to a broad spectrum of materials, including polymers, semiconductors and magnetic materials. This synthetic methodology can be further adopted as a new experimental platform for designing and rapidly prototyping functional colloids, such as reconfigurable micro swimmers, colloidal surfactants and switchable building blocks for self-assembly.

## Abstract (web summary)

Mimicking the intrinsic adaptability of biological systems in synthetic materials has been a challenge. Here, Sacanna and co-workers have used dewetting forces between an oil phase and solid colloidal substrate to facilitate shape shifting particles that can change geometry by chemical and optical signals.

## Graphical abstract

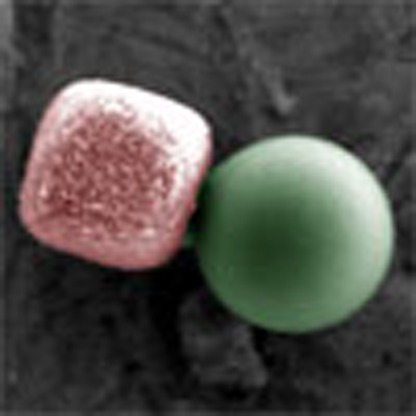

At all length scales, shape alone can set the path for the spontaneous self-organization of elementary units into remarkably complex architectures^1^. The programmed structural rearrangements of proteins, for instance, drive a broad range of important functions from molecular recognition, sensing and reporting, to macroscopic changes in the optical and mechanical bulk properties of biomaterials. Minute morphological variations in the shape of the building blocks can tip the balance towards a desired structure or random aggregate^2^. At the nano- and micron-scale, this concept is particularly intriguing since the self-assembly of rationally designed colloidal building blocks could lead to the next generation of functional materials. This realization has given rise to a new and rapidly growing form of material engineering that aims at using self-assembly of colloidal matter as a nano-fabrication process^3^,^4^. An enormous theoretical and computational effort has taken place to determine the optimal attributes of building blocks for target structures and to extract general design principles for self-assembly^5^,^6^,^7^. At the same time, advances in synthetic colloidal chemistry have enabled us to shape particles into new geometries^8^,^9^, and to tailor their interactions by means of entropic^10^,^11^, chemical^12^,^13^ and non-equilibrium forces^14^,^15^. As a result, extensive colloidal architectures, with increasing structural complexity, are shown to emerge spontaneously from Browninan suspensions of elementary building blocks. In contrast to biological matter, however, the majority of these self-assembled materials are intrinsically static, which limits the control over structural changes to externally applied fields or variations in the interaction potential between the constituent building blocks.

Currently, the main strategy to impart shape-changing abilities to colloidal particles is to utilize stimuli responsive polymers (typically thermo- or pH-responsive). These materials have been cleverly used to cause anisotropic swelling in colloidal particles, thus yielding spheroids with adjustable aspect ratios^16^,^17^,^18^,^19^.

In this work, we describe a new approach based on dewetting forces. We demonstrate that solid particles engulfed in oil droplets can be controllably and reproducibly dewetted by chemically reducing the oil's affinity towards the substrate, thus yielding new colloidal morphologies. Using this principle, we create a whole new class of shape-shifting particles whose geometries can be dynamically changed using chemical or light stimuli.

## Results

### Homogeneous nucleation of reactive emulsions

Our synthetic methodology can be summarized in a few basic steps: we start with a suspension of hydrophilic solid particles (the seeds); we then covalently attach silane molecules to the seed's surface to create an hydrophobe monolayer; next the hydrophobized seeds are engulfed in oil droplets; and finally the hydrophobe layer is chemically degraded to allow a controllable dewetting of the seed towards new equilibrium morphologies. We will now describe each step in detail. The nucleation of monodispersed oil droplets from a homogeneous aqueous solution can be achieved via the base-catalysed condensation of alkoxysilanes into insoluble silsesquioxane oligomers^20^. This emulsification mechanism can be rationalized as follows. Initially, a tri-alkoxysilane (the oil precursor) is added to an aqueous solution containing ammonia. In this chemical environment, the labile alkoxy groups of the silane monomer are hydrolyzed to form water-soluble silanols, which rapidly condense into silsesquioxane oligomers. As these oligomers grow larger, their solubility in the water phase decreases and they eventually phase separate. Interestingly, however, instead of two macroscopic phases, this phase separation leads to highly monodispersed oil droplets, which form via a classic nucleation and growth mechanism (Supplementary Fig. 1 and Supplementary Methods). This can be understood by considering that the newly formed oil phase contains silanol moieties that can undergo deprotonation at the oil–water interface, thus providing electrostatic stabilization for the growing oil droplets, which prevents their coalescence. This surface charge manifests itself with a negative *ζ*-potential of about −30 mV at a pH of 7, which monotonically decreases to a value of about −80 mV at a pH of 12 (Supplementary Fig. 2 and Supplementary Methods).

### Heterogeneous nucleation

**Figure 1:**
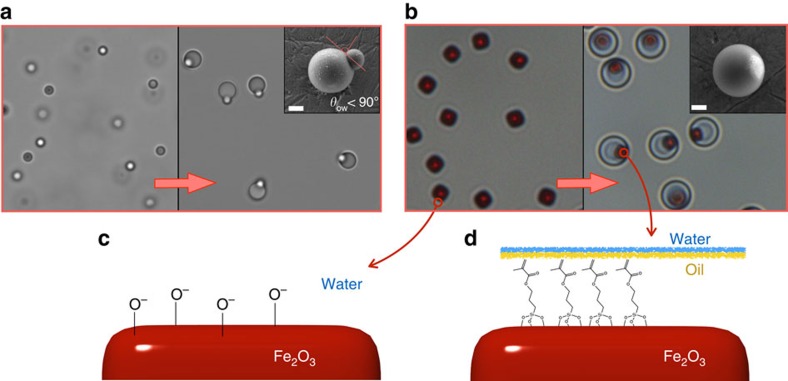
Heterogeneous nucleation of silicon oil on different solid seed particles. (**a**) When the TPM oil precursor is added to a colloidal suspension of negatively charged polystyrene spheres, the heterogeneous nucleation and growth of oil droplets occurs on the solid seed particles, resulting in a well-defined contact angle between the seed particle and the oil phase. (**b**) However, when the TPM oil precursor is added to a suspension of haematite cubes, we observe the complete encapsulation of the seeds inside the oil phase. The inserts show SEM images of the polymerized hybrid particles. Scale bars, 800 nm. We explain this behaviour with the formation of a hydrophobic layer of silane molecules that condense and covalently bind to the haematite substrate. (**c**,**d**) Schematics showing the haematite surface chemistry before and after the formation of the hydrophobizing silane layer.

When solid impurities are present, the formation of the oil phase occurs via heterogeneous nucleation, generating nano- and micron-sized oil-in-water droplets carrying a single solid inclusion. We illustrate this point in Fig. 1a by using monodispersed polystyrene spheres as model impurities. Each polystyrene seed particle triggers the nucleation of an oil droplet, which partiality wets the seed with a well-defined contact angle. Interestingly, when haematite (*α*-Fe_2_O_3_) colloids are used as the seeds, we observe an unexpectedly high wettability, which results in the seeds being fully encapsulated in the oil droplets (Fig. 1b). Given the marked hydrophilicity of the haematite seeds at the reaction conditions (*ζ*≈−40 mV at pH=9), this result seems, at first, rather unusual. However, one must consider that the silanols produced during the initial stages of the oil nucleation are highly reactive and readily form covalent bonds with hydroxyl-containing substrates. This leads to the formation of a silane hydrophobe layer where the Si moiety of the oil is bound to deprotonated oxygen atoms on the seed particles, effectively making their surface hydrophobic (Fig. 1c,d). To confirm this hypothesis, encapsulated seeds were recovered from the oil phase by a repeated washing treatment in ethanol (in which the oil is highly soluble), and then brought back to water to assess their colloidal stability. Even after several washing steps, the recovered seeds are no longer stable in the aqueous medium, but instead they rapidly aggregate into macroscopic sediments (Supplementary Fig. 3 and Supplementary Methods). A gentle etching treatment using hydrochloric acid, however, is sufficient to erode a thin surface layer of the substrate and the corresponding silane molecules bound to it. As expected, this treatment fully restores the original particle's hydrophilicity and yields a stable suspension.

### Reconfigurability via stimulated dewetting

**Figure 2:**
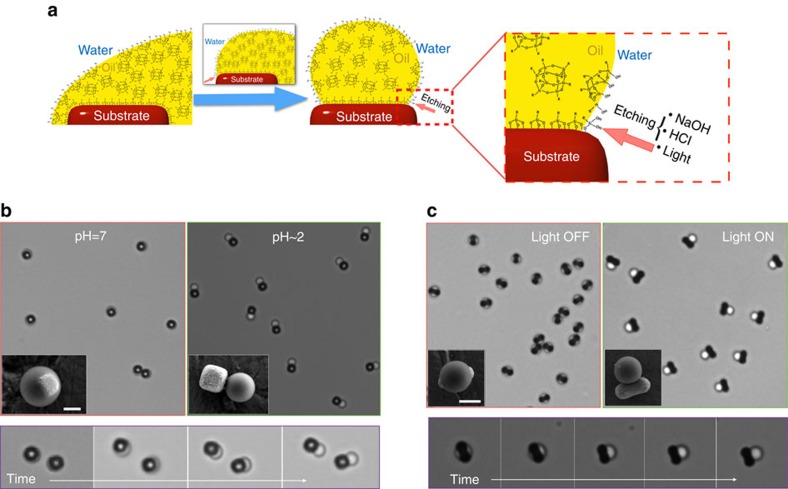
pH- and light-triggered dewetting. (**a**) Cartoon illustrating how the oil phase gradually dewets from the substrate as a consequence of the chemical etching of the silane hydrophobe monolayer. Depending on the type of substrate, the dewetting can be triggered by NaOH, HCl or light. Two different silane-based oil phases were used in our experiments: TPM, which can be hardened via radical polymerization, and 3-(chloropropyl)trimethoxysilane, which self-hardens in the presence of NH_3_. (**b**,**c**) Optical microscopy images showing the practical realization of shape-shifting particles using the mechanism described in **a**. In **b** we used colloidal cubes as substrates and we triggered the dewetting by a pH change. In **c** we used peanut-shaped substrates and exploited their photocatalytic properties to realize a light-triggered dewetting mechanism. For each sample, the images show a large field of view before and after the dewetting stimulus, a timelapse of the dewetting dynamics, and SEM pictures of hardened particles before and after reconfiguration. All scale bars, 1 μm. See the corresponding Supplementary Movies 1 and 2.

**Figure 3:**
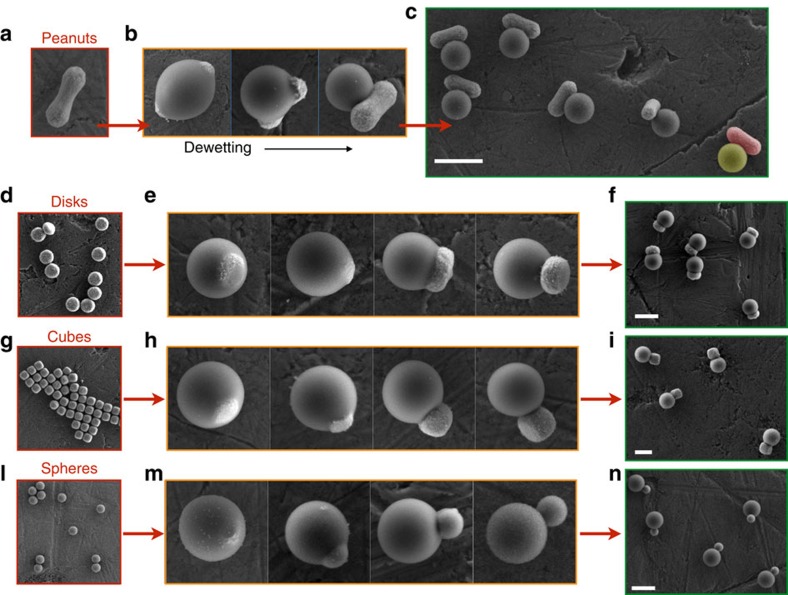
Fabrication of reconfigurable colloids. (**a**) A seed suspension of haematite colloids is used to nucleate liquid silsesquioxane micro-droplets that fully encapsulate the seeds. (**b**) The addition of NaOH coupled with heat promotes the dewetting of the oil from the seeds. (**c**) The new morphology of the particles is fixed via a radical polymerization, resulting in a suspension of fully solid colloids. In **c** the particle at the bottom right has been false-coloured to illustrate its hybrid nature; red indicates the haematite seed and yellow the polymerized oil. We have applied our synthetic methodology to seeds of various shapes and compositions. (**d**,**g**,**l**) Disc-like haematite particles, cubic haematite particles and spherical silica particles, respectively, used as seeds for heterogeneous nucleation. (**e**,**h**,**m**) Each sequence represents a timelapse capturing the dewetting of the seeds and the corresponding morphological change. (**f**,**i**,**n**) SEM images showing larger field of views to illustrate the homogeneity of the samples. All scale bars, 2 μm.

Next, we exploit this encapsulation mechanism for producing reconfigurable hybrid emulsions, as well as colloidal building blocks with shapes that were previously inaccessible. The idea, schematized in Fig. 2a, is to induce the dewetting of the oil droplets from the seeds by chemically etching the oil-substrate siloxane linkages that are responsible for the formation of the passivating hydrophobic layer. To this end, we demonstrate several viable methods that can be used to achieve this goal, depending on the nature of the seed particles used. For haematite substrates, the most obvious way to stimulate the dewetting of the oil, is to simply repeat the same etching treatment in HCl that we used previously to restore the particles hydophilicity. This is shown in Fig. 2b, where we lowered the pH of the emulsion to about 2 to observe a radical change in the particles morphology (Supplementary Movie 1). Interestingly, we noticed that the dewetting pathways strongly depend on the geometry of the seeds, and for identically shaped seeds our dewetting scheme yields highly reproducible shapes as we illustrate in Figs 2c and 3. Larger field of views are shown in Supplementary Fig. 4.

While the etching of the substrate works particularly well with haematite seeds, a more general chemical trigger for dewetting is the addition of NaOH. Suspending encapsulated seeds in a solution of 60 mM NaOH and gently raising the temperature to 65 °C, in fact, causes the siloxane bridges to undergo to a rapid hydrolytic decomposition independently from the substrate used during encapsulation. This, once again, weakens the adhesion force between the oil and the substrate and prompts the dewetting of the seeds in a similar fashion as for the HCl treatment. As we show in Fig. 3, we have applied this base-triggered dewetting process to haematite, silica and titania seeds, obtaining nearly identical results.

### Photo-stimulated dewetting

A third and most intriguing dewetting mechanism is offered by a modified photo-Fenton reaction^21^, which takes advantage of the photocatalytic properties of the colloidal substrate to degrade the silane hydrophobe layer. While this mechanism only works with photo-active substrates, we found it particularly interesting as the particles can remain in their native aqueous medium at a neutral pH and be dewetted by simply exposing them to light. For haematite seeds, light-stimulated dewetting was realized by irradiating the sample with green or blue light (*λ*≤560 nm) in the presence of a small amount of hydrogen peroxide (typically ≤2.5% wt). The irradiation promotes the formation of hydroxyl radicals (OH·), which readily degrade the hydrophobe layer, revealing the bare haematite surface to the oil. This is demonstrated by the time-lapse shown in Fig. 2c, where initially spherical particles respond to the light input by rapidly reconfiguring into new equilibrium morphologies (Supplementary Movie 2). Depending on the experimental conditions, such as light intensity and H_2_O_2_ concentration, this reconfiguration can take from a few seconds to minutes to complete. Interestingly, at high light intensities the oil droplets can be completely detached from the seeds.

### Generalization of the mechanism

Because alkoxysilanes can form stable condensation products with many different oxides such as those of titanium, aluminum, zirconium, tin, iron and nickel, we expect this reconfiguration mechanism to be quite easily extended to many other colloidal systems. We have tested seeds of various shapes and compositions, including haematite, titania and silica, and have observed nearly identical dewetting dynamics; some of these results are summarized in Fig. 3.

**Figure 4:**
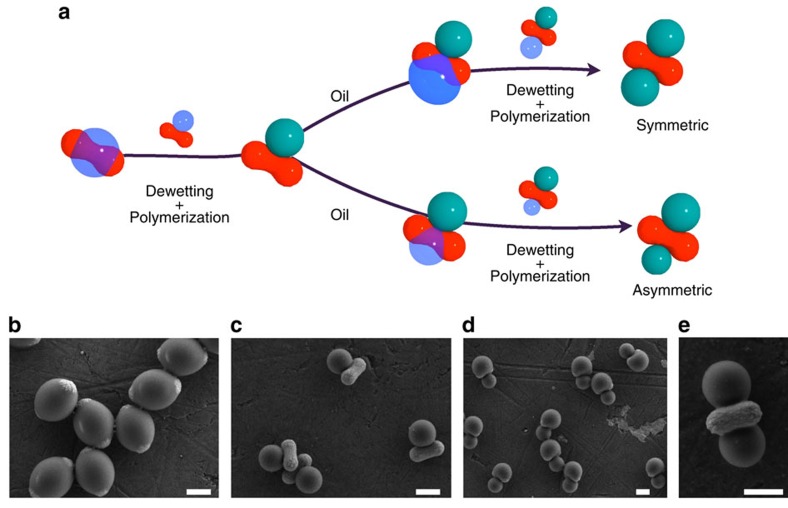
Engineering new shapes. (**a**) A schematic showing the oil-nucleation, dewetting and polymerization process. Dewetted particles can be further processed to produce shape-anisotropic colloids by controlling the amount of TPM oil precursor added to the dewetted particles. (**b**) SEM image showing the encapsulation of peanut-like haematite seeds in the TPM oil phase. (**c**) SEM image showing uniform dewetted particles. (**d**) SEM image showing the particles from **c** being used as seeds to grow TPM on the exposed haematite surface. (**e**) SEM image showing the particles in **d** after iterating the dewetting and oil polymerization steps. All scale bars, 1 μm.

Figure 3 also illustrates an interesting feature of one of the silane compounds utilized in this study, namely its ability to be chemically hardened via a free radical polymerization. When 3-(trimethoxysilyl)propyl methacrylate (TPM) is used as the oil phase, a radical initiator can cause the crosslinking of the silsesquioxane oligomers through their methacrylate moieties, thus effectively solidifying the oil into an organo-silica hybrid. This polymerization enables us to study the reconfiguration dynamics in great detail by rapidly fixing the intermediate morphologies throughout the dewetting process, and subsequently imaging these states by scanning electron microscopy (SEM). After polymerization, the final solid product can be dried, resuspended and further processed. For example, the process of oil nucleation, dewetting and polymerization can be repeated to rationally install multiple reconfigurable lobes on the same seed particle. This is demonstrated in Fig. 4, where the anisotropic particles shown in Fig. 3c are reutilized as seeds in a second oil-nucleation step. As expected, the newly nucleated oil wets the exposed haematite, avoiding the preexisting lobe due to its stronger surface charge and hydrophilic character (Supplementary Figs 5 and 6 and Supplementary Methods). This particle's new geometry can now be fixed by a second free radical polymerization or further reconfigured by a subsequent dewetting step to obtain the shape shown in Fig. 4e. The versatility of this synthetic scheme relies on the fact that the colloidal design is here reduced to a wetting problem, thus allowing the *a priori* engineering of complex colloidal shapes by a rational choice of the solid substrate and the oil phase.

### Reconfigurable colloidal architectures

**Figure 5:**
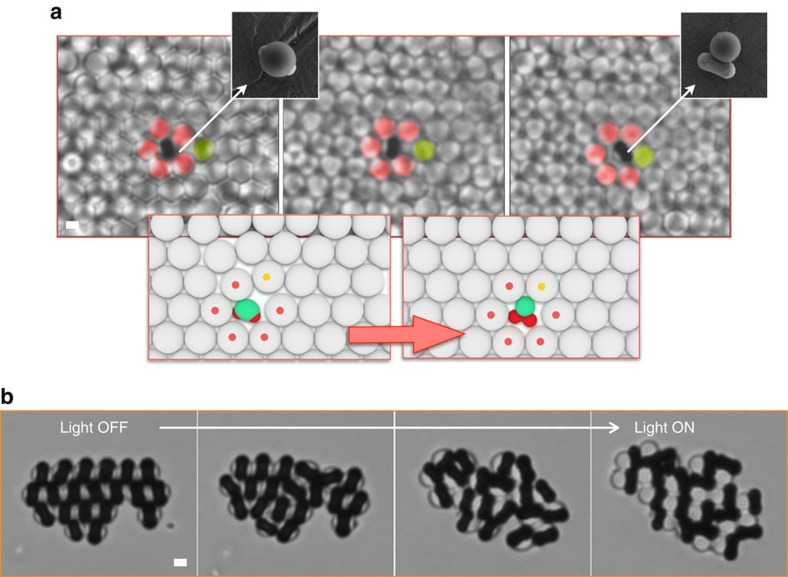
Reconfigurable materials. (**a**) Timelapse (from Supplementary Movie 3) showing colloidal spheres arranged in a two-dimensional hexagonal lattice. In the centre of the lattice, a substitutional impurity consisting of a smaller shape-shifting particle is creating a visible strain in the lattice. The strain disappears when the substitutional particle reconfigures into its new morphology. (**b**) Shape-shifting particles are first assembled by depletion forces into a colloidal crystallite and then allowed to reconfigure under a light stimulus. The timelapse (from Supplementary Movie 4) shows how the whole crystal gradually morphs into a new striped microstructure. All scale bars, 1 μm.

While shape alone has been frequently used to drive self-assembly to generate complex organization, the use of building blocks with dynamically reconfigurable geometries is yet to be experimentally explored. We believe that this new family of shape-shifting colloids might provide unique opportunities for the topological control of structures in a dynamic manner, with ordered, quasi-ordered and network-like morphologies that can be switched on demand. For example, the incorporation of shape-shifting building blocks into self-assembled colloidal architectures could be used to induce local structural rearrangements, bulk phase changes or to engineer switchable lattice defects. We explore this idea with two experiments that we summarize in Fig. 5. In the first experiment (Fig. 5a) we assembled a colloidal crystal doped with a small amount of reconfigurable colloids acting as substitutional impurities. Initially, the odd shape of the substitutional particles causes a visible distortion in the host lattice, however, as the particles reconfigure into their new geometry the lattice rearranges and the strain disappears (Supplementary Movie 3). This idea could be further developed to engineer switchable line- or dislocation-like defects within microstructured materials, to enable, for example, healing or controlled failures.

In the second experiment, we demonstrate how the reconfiguration of an entire colloidal crystal can lead to architectures that would normally be inaccessible via direct self-assembly. The timelapse of Fig. 5b (taken from Supplementary Movie 4) shows a crystallite assembled by depletion forces that, on exposure to blue light, reconfigures into a striped phase consisting of alternating oil droplets (light spheres) and elongated iron-oxide particles (black particles). This striped microstructure would normally be inaccessible because of the magnetic attraction between the iron oxide particles that would segregate them into a dense cluster.

## Discussion

We have demonstrated that chemically triggered wetting–dewetting phenomena can be utilized to engineer shape-shifting colloids with addressable geometries and compositions. This synthetic concept allowed us to design unusually complex colloidal shapes and reproducibly synthesize them in bulk amounts. Because the method is general and applicable to broad spectrum of materials, it can serve as a fast prototyping platform for the preparation of the next generation of non-spherical light-activated swimmers, active Janus surfactants and shape-shifting particles. The reconfigurability, in particular, can open new avenues for fabricating bulk microstructured materials from self-assembled colloidal crystals and enable us to find new rules for geometry-driven assembly processes. An interesting challenge for the future is to engineer smarter hydrophobe layers that could be set on or off on demand. This would allow for the reversibility of the shape shifting and enable, for example, the assembly of switchable devices.

## Methods

### Synthesis of the seed particles

Monodisperse haematite cubes and peanuts are prepared via the gel–sol method previously described by Sugimoto^22^,^23^. Briefly, haematite cubes are prepared by mixing 100 ml of 2 M FeCl_3_·6H_2_O, 90 ml 6 M NaOH and 10 ml water thoroughly (for peanuts, replace the final 10 ml water in the aforementioned mixture with 10 ml of 0.6 M Na_2_SO_4_). The gel formed is aged at 100 °C for 8 days in a sealed 250-ml Pyrex bottle. The final product was isolated by repeated sedimentation and resuspension cycles in deionized water (Supplementary Fig. 7 and Supplementary Methods). Silica seeds were prepared following the Stöber method^24^, while titania seeds were prepared following the method reported by Tanaka *et al*.^25^.

### Homogeneous nucleation of oil droplets

The homogeneous nucleation of monodispersed oil droplets can be obtained by adding 1.5 ml of TPM (≥98%, from Sigma-Aldrich) to a solution containing 300 μl of NH_3_ (28% wt) in 300 ml deionized water under stirring. Once growth has ceased, which can be monitored via optical microscopy and typically takes about 1 h, the resulting spherical emulsion droplets are about 1 μm in diameter. The final droplet size can be changed by either varying the ammonia concentration or the initial amount of oil precursor. Alternatively, 3-(chloropropyl)trimethoxysilane (≥97%, from Sigma-Aldrich) can be used as an oil precursor following the same procedure described for TPM.

### Heterogeneous nucleation of oil droplets on solid seeds

To encapsulate haematite seeds into oil droplets, 60 μl of NH_3_ (28% wt) is added to 60 ml aqueous suspension of haematite particles (about 0.5% wt), followed by the addition of 400 μl of the oil precursor (TPM or 3-(chloropropyl) trimethoxysilane). The mixture is kept under stirring and the growth can be monitored at regular intervals via optical microscopy. Additional oil precursor can be added to the suspension (200 μl for each subsequent addition) once growth has ceased to reach a desired particle size.

Under the same basic oil-nucleation conditions, silica carries a stronger negative charge than haematite, making it a poor host for the nucleation of the oil onto its surface. Indeed, attempting to grow the oil on bare silica seeds always results in the homogeneous nucleation of oil droplets that coexist with the seed particles in a stable bidispersed suspension. To nucleate the oil onto Stöber silica, a small amount of a cationic surfactant (0.01 mM of dodecyl trimethyl ammonium bromide, ≥97%, from Sigma-Aldrich) is added to the silica suspension (Supplementary Fig. 8 and Supplementary Methods). More details about the heterogenous nucleation of oil droplets are given in Supplementary Fig. 9 and Supplementary Methods.

### Dewetting experiments

To improve the stability of the oil droplets and prevent their coalescence during the etching treatments, Pluronic F-108 is added to the emulsions to a final concentration of ∼0.01% wt. Dewetting is achieved in 60 mM NaOH while heating at 65 °C under mild mechanical stirring. Alternatively, the dewetting of oil from haematite seeds was carried out by adding HCl to a final concentration of about 50 mM. To dewet photoactive substrates, the particles were suspended in 2.5% wt H_2_O_2_ and irradiated with a blue light from a mercury lamp source (Leica EL6000) coupled to an inverted microscope (Leica DMI3000). The extent of dwetting can be controlled by tuning the amount of time the particles are exposed to the light. Prolonged exposure results in the complete detachment of the oil droplet from the seed particles. The polymerization of the TPM-based particles is carried out by adding about 0.5 mg of 2, 2′-Azobis(2-methylpropionitrile) (98%, from Sigma-Aldrich) to the suspension and heating the mixture at 80 °C for 3 h. The polymerized particles are then typically cleaned by a two cycles of sedimentation and resuspension in deionized water. See also Supplementary Methods for additional details on dewetting experiments.

### Reconfigurable crystals

Colloidal crystals were assembled inside borosilicate glass capillaries (inner dimensions 2.0 × 0.1 mm, Vitrocom) using depletion interactions. We used Ludox (HS-40, from Sigma-Aldrich) silica nanoparticles as the depletant at a concentration of typically 6% wt. Haematite particles are first encapsulated into oil droplets via heterogeneous nucleation and are then resuspended in deionized water via two cycles of sedimentation and resuspension. The cleaned particles are then suspended in 2.5% wt H_2_O_2_ and 6% wt Ludox HS-40 in a sealed glass capillary. The glass capillary is then left undisturbed for about 1 h to allow the particles to crystallize. Once the colloidal crystals have formed, reconfiguration is achieved by simple exposure to blue light.

### Imaging

Both bright field and fluorescent microscopy images and videos were acquired using a Leica DMI3000 inverted microscope equipped with DIC optics and high-resolution CMOS camera (Hamamatsu ORCA Flash4.0 sCMOS). Electron microscopy images were takes using a MERLIN (Carl Zeiss) field emission SEM. Before imaging, colloidal suspensions were dried on SEM stubs and analysed without any further treatment.

### Thermogravimetric analysis

An interesting property of alkoxysilanes is their ability to be thermally decomposed (calcinated) to SiO_2_. Thermogravimetric analysis on polymerized TPM shows that above 700 °C, the organic matter in the silsesquioxane network burns off leaving a denser silica network. As a result, polymerized TPM particles shrink to about 17% of their original volume. The effect of the thermal treatment is evident by comparing SEM of samples before and after the heat treatment (Supplementary Fig. 10). Thermogravimetric analysis was performed with a PerkinElmer Pyris 1 TGA thermogravimetric analyser.

### Density measurements

Density measurements were performed using an Anton-Paar DMA 4500M Digital Density Meter. We analysed unpolymerized TPM oil droplets, polymerized TPM particles and calcinated TPM particles, and found their densities to be 1.23, 1.31, and 2.08 g cm^−3^, respectively (Supplementary Fig. 10).

### Data availability

The data that support the findings of this study are available from the corresponding author on request.

## Additional information

**How to cite this article:** Youssef, M. *et al*. Shape-shifting colloids via stimulated dewetting. *Nat. Commun.* 7:12216 doi: 10.1038/ncomms12216 (2016).

## Supplementary Material

Supplementary Information Supplementary Figures 1-10, Supplementary Methods, and Supplementary References

Supplementary Movie 1 Dewetting of oil droplets from Hematite seeds using HCl. Video microscopy showing the HCl-triggered dewetting of oil from colloidal cubes of Hematite. Video played at 10X real time.

Supplementary Movie 2 Dewetting and detachment of oil droplets from Hematite seeds using light. Video microscopy showing the light-triggered dewetting of oil from peanut-shaped Hematite colloids. Video played at 3X real time.

Supplementary Movie 3 Switchable defect. Video microscopy showing a colloidal crystal doped with a shape-shifting particle acting as a point defect. When the crystal is illuminated, the impurity change shape, thus causing the lattice to rearrange and the strain disappears.

Supplementary Movie 4 Light-reconfigurable colloidal crystal. Video microscopy showing a reconfigurable colloidal crystal (assembled by depletion forces from individual shape-shifting building blocks) that morphs into a striped microstructure after being exposed to light. Video played at 3.7X real time.

## References

[b1] Whitesides G. M. Self-assembly at all scales. Science 295, 2418–2421 (2002). 11923529 10.1126/science.1070821

[b2] Rossi L. . Shape-sensitive crystallization in colloidal superball fluids. Proc. Natl Acad. Sci. USA 112, 5286–5290 (2015). 25870301 10.1073/pnas.1415467112 PMC4418869

[b3] Cademartiri L., Bishop K. J. M., Snyder P. W. & Ozin G. A. Using shape for self-assembly. Phil. Trans. R. Soc. A 370, 2824–2847 (2012). 22615463 10.1098/rsta.2011.0254

[b4] Ozin G. A. . Nanofabrication by self-assembly. Mater. Today 12, 12–23 (2009).

[b5] Damasceno P. F., Engel M. & Glotzer S. C. Predictive self-assembly of polyhedra into complex structures. Science 337, 453–457 (2012). 22837525 10.1126/science.1220869

[b6] van Anders G., Ahmed N. K., Smith R., Engel M. & Glotzer S. C. Entropically patchy particles: engineering valence through shape entropy. Acs Nano 8, 931–940 (2014). 24359081 10.1021/nn4057353

[b7] Nguyen T. D., Jankowski E. & Glotzer S. C. Self-assembly and reconfigurability of shape-shifting particles. Acs Nano 5, 8892–8903 (2011). 21950837 10.1021/nn203067y

[b8] Glotzer S. C. & Solomon M. J. Anisotropy of building blocks and their assembly into complex structures. Nat. Mater. 6, 557–562 (2007). 17667968 10.1038/nmat1949

[b9] Sacanna S., Pine D. J. & Yi G.-R. Engineering shape: the novel geometries of colloidal self-assembly. Soft Matter 9, 8096 (2013).

[b10] Sacanna S., Irvine W. T. M., Chaikin P. M. & Pine D. J. Lock and key colloids. Nature 464, 575–578 (2010). 20336142 10.1038/nature08906

[b11] Kraft D. J. . Surface roughness directed self-assembly of patchy particles into colloidal micelles. Proc. Natl Acad. Sci. USA 109, 10787–10792 (2012). 22715288 10.1073/pnas.1116820109 PMC3390863

[b12] Macfarlane R. J., Jones M. R., Lee B., Auyeung E. & Mirkin C. A. Topotactic interconversion of nanoparticle superlattices. Science 341, 1222–1225 (2013). 23970559 10.1126/science.1241402

[b13] Wang Y. . Colloids with valence and specific directional bonding. Nature 490, 51–55 (2012). 23128225 10.1038/nature11564

[b14] Palacci J., Sacanna S., Steinberg A. P., Pine D. J. & Chaikin P. M. Living crystals of light-activated colloidal surfers. Science 339, 936–940 (2013). 23371555 10.1126/science.1230020

[b15] Sengupta S., Ibele M. E. & Sen A. Fantastic voyage: designing self-powered nanorobots. Angew. Chem. Int. Ed. 51, 8434–8445 (2012). 10.1002/anie.201202044 22887874

[b16] Tu F. & Lee D. Shape-changing and amphiphilicity-reversing janus particles with pH-responsive surfactant properties. J. Am. Chem. Soc. 136, 9999–10006 (2014). 24791976 10.1021/ja503189r

[b17] Klinger D. . A facile synthesis of dynamic, shape-changing polymer particles. Angew. Chem. Int. Ed. 53, 7018–7022 (2014). 10.1002/anie.201400183 PMC4074252 24700705

[b18] Yang Z., Huck W. T. S., Clarke S. M., Tajbakhsh A. R. & Terentjev E. M. Shape-memory nanoparticles from inherently non-spherical polymer colloids. Nat. Mater. 4, 486–490 (2005). 15895098 10.1038/nmat1389

[b19] Petrov P. G. & Döbereiner H. G. in Perspectives in Supramolecular Chemistry: Giant Vesicles eds P. L. Luisi & P. Walde) Vol. 6, (John Wiley & Sons, Ltd., Chichester, UK (2000).

[b20] Obey T. & Vincent B. Novel monodisperse ‘silicone oil'/water emulsions. J. Colloid Interface Sci. 163, 454–463 (1994).

[b21] Castillo S. I. R. . Colloidal cubes for the enhanced degradation of organic dyes. J. Mater. Chem. A 2, 10193–10199 (2014).

[b22] Sugimoto T., Khan M. M. & Muramatsu A. Preparation of monodisperse peanut-type alpha-Fe2o3 particles from condensed ferric hydroxide gel. Colloids Surf. A 70, 167–169 (1993).

[b23] Sugimoto T., Itoh H. & Mochida T. Shape control of monodisperse hematite particles by organic additives in the gel–sol system. J. Colloid Interface Sci. 205, 42–52 (1998). 9710498 10.1006/jcis.1998.5588

[b24] Stober W., Fink A. & Bohn E. Controlled growth of monodisperse silica spheres in micron size range. J. Colloid Interface Sci. 26, 62–6 (1968).

[b25] Tanaka S., Nogami D., Tsuda N. & Miyake Y. Synthesis of highly-monodisperse spherical titania particles with diameters in the submicron range. J. Colloid Interface Sci. 334, 188–194 (2009). 19398105 10.1016/j.jcis.2009.02.060

