# Supplementary material for: Shape-shifting colloids via stimulated dewetting
Source: Nat Commun. 2016 Jul 18;7:12216. doi: 10.1038/ncomms12216 (PMC4960307; doi:10.1038/ncomms12216)
Supplement: Supplementary Information — Supplementary Figures 1-10, Supplementary Methods, and Supplementary References [file ncomms12216-s1.pdf]

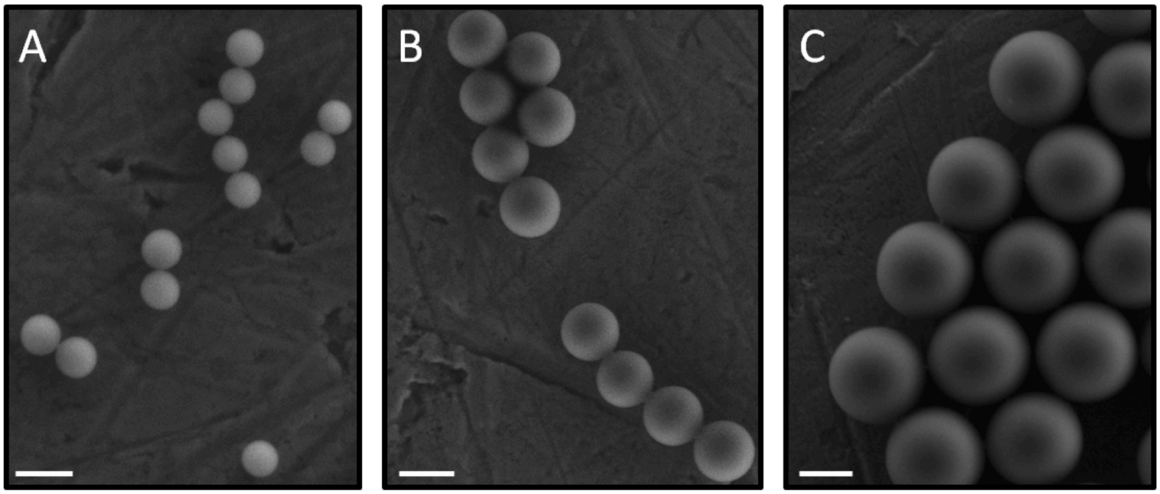

Supplementary Figure 1 SEM images of polymerized oil droplets (*p*-TPM) synthesized by varying the concentrations of ammonia and TPM precursor. (A) 220 nm, (B) 520 nm, and (C) 1300 nm. Scale bars are 300 nm, 500 nm, and 700 nm, respectively

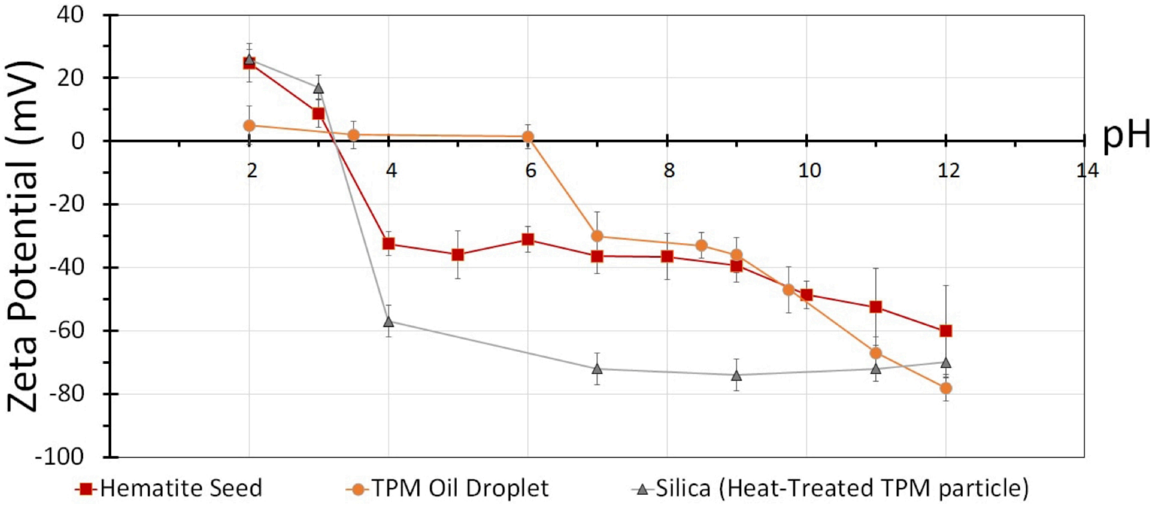

Supplementary Figure 2 Zeta potential vs. pH for hematite seeds, unpolymerized TPM oil droplets, and calcinated p-TPM particles. Lines connecting data points are used to guide the eye and are not representative of collected data.

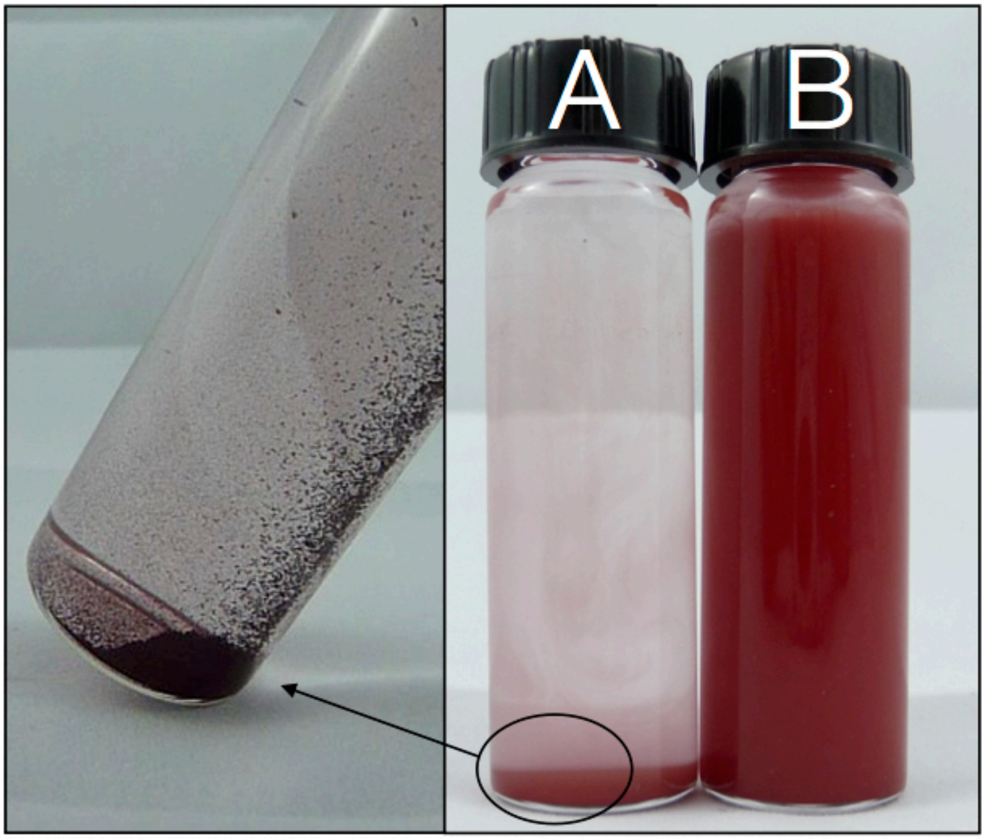

Supplementary Figure 3 Hematite seeds that have been coated with TPM, washed in ethanol and resuspended in deionized water (left) form aggregates, are unstable, and quickly sediment (A). Bare hematite particles that are subject to identical washes in ethanol and deionized water are stable and remain suspended in both solvents (B).

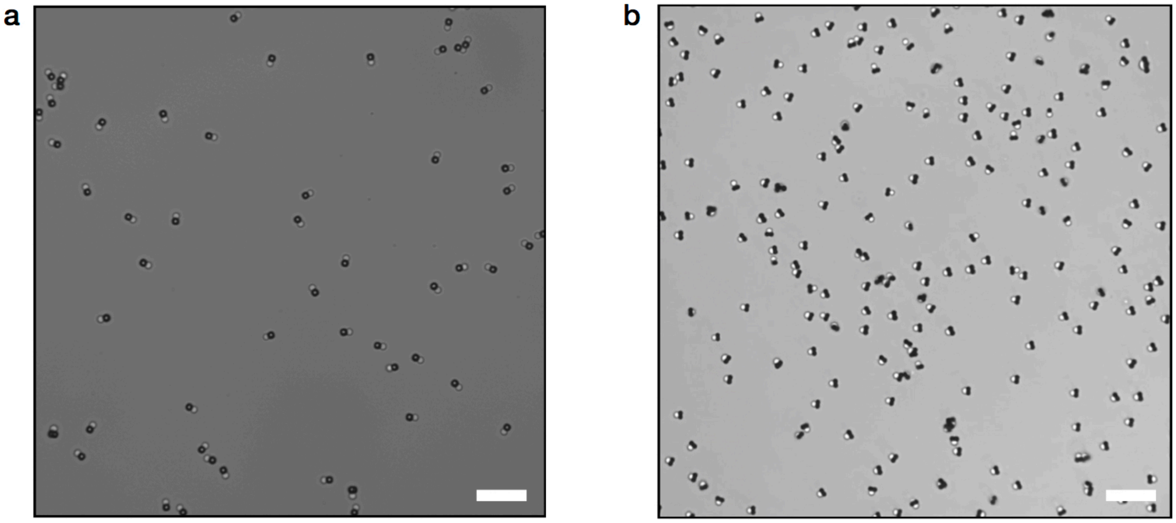

Supplementary Figure 4 Bright-field optical microscopy images showing large collections of dewetted particles. (a) Dewetted cubic seeds. (b) Dewetted peanut-shaped seeds. Scale bars are 10 μm.

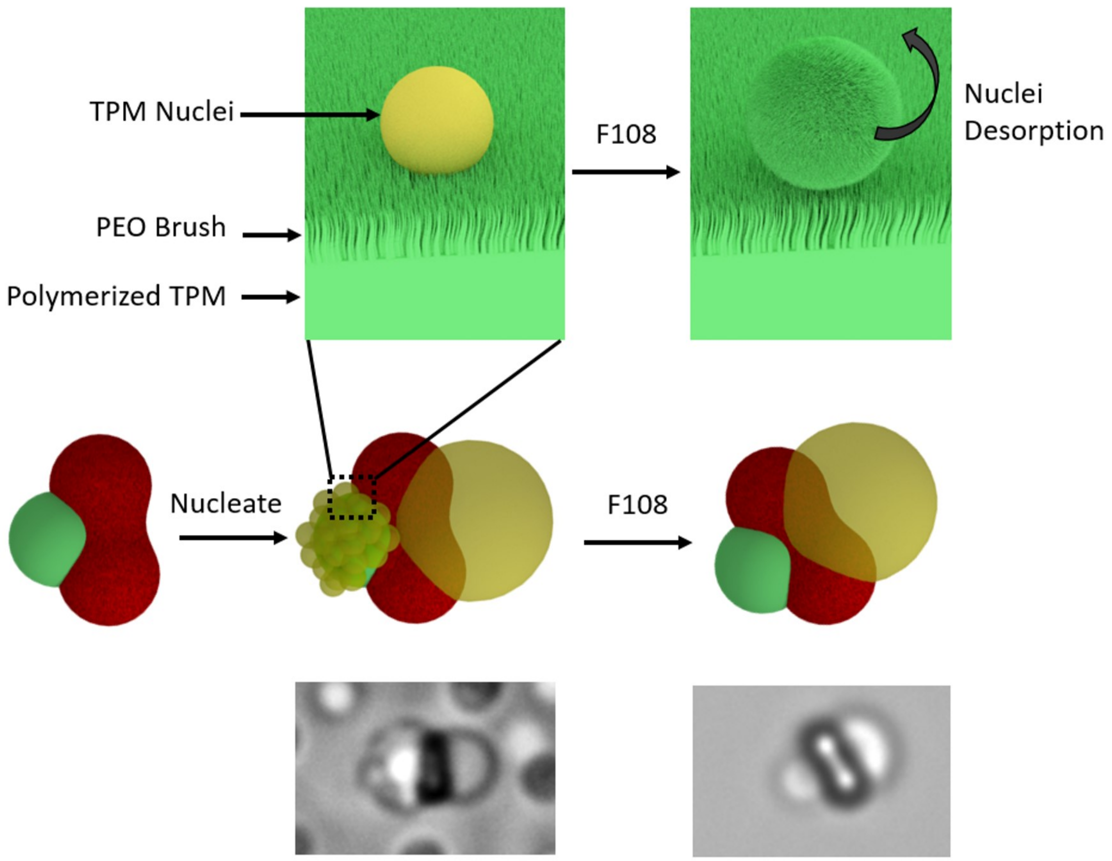

Supplementary Figure 5 Nucleation of TPM on dewetted particles results in two types of growth. Secondary TPM oil droplets are physisorbed on the p-TPM-f108 and can be liberated with the addition of surfactant. TPM that nucleates and grows on hematite is chemisorbed and remains even after the addition of surfactant (see Supplementary Methods).

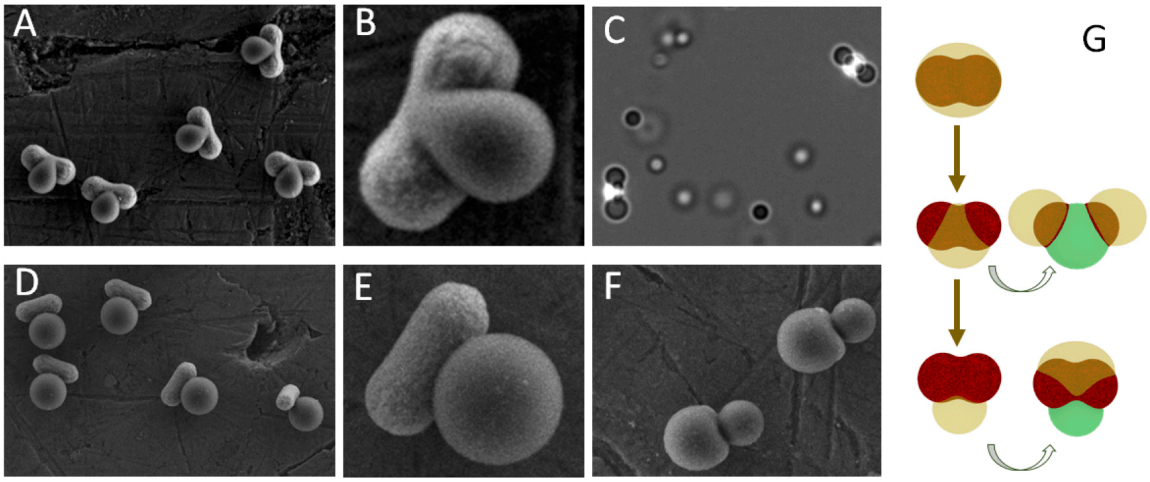

Supplementary Figure 6 The extent of dewetting greatly influences how TPM will grow on dewetted particles. When TPM is not fully dewetted from the hematite seed particles (A,B) TPM grows on two distinct hematite surfaces (C). The TPM from the first growth is fluorescently labeled to show that dewetting did not proceed to completion. When TPM is fully dewetted from the seeds (D,E), TPM forms a single lobe on the hematite surface (F). (G) shows a schematized depiction of both scenarios.

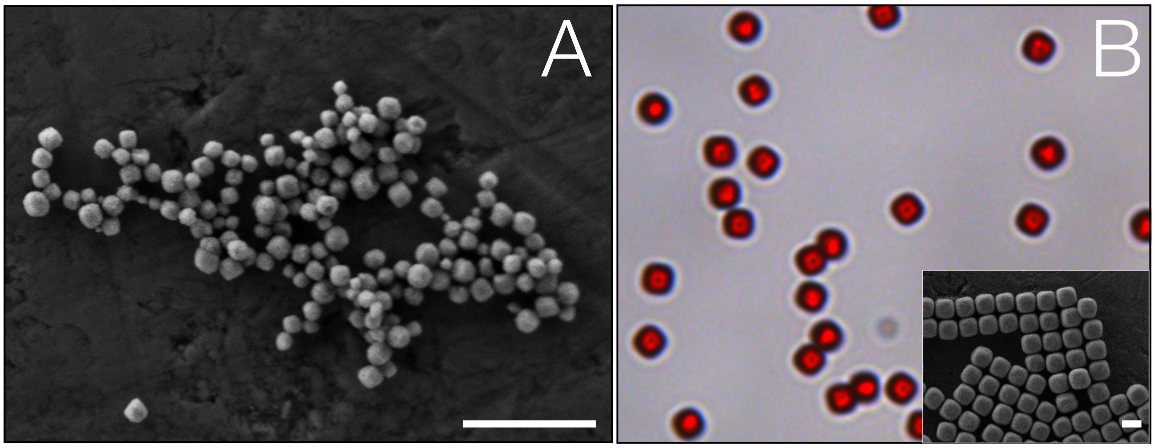

Supplementary Figure 7 (A) An SEM image of small and polydispersed hematite particles produced due to incorrect $FeCl_3$ concentration (Supplementary Methods). Scale bar is 300 nm. (B) Monodispersed hematite cubes synthesized by accounting for absorbed moisture in the FeCl3 powder. Inset shows an SEM image of the hematite cubes. Scale bar is 1 μm.

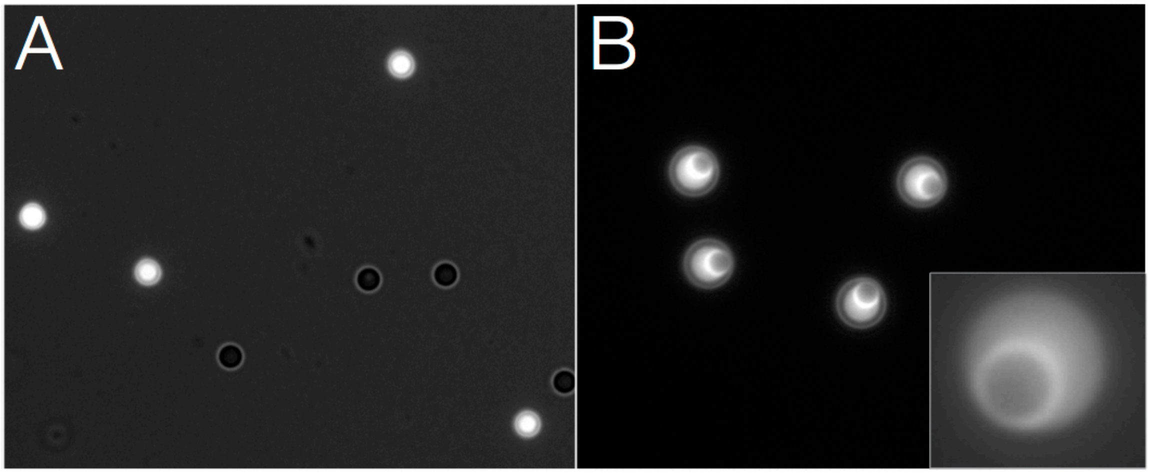

Supplementary Figure 8 (A) TPM oil added to a basic suspension of silica particles results in TPM nucleating and growing separate from silica. The fluorescent particles are TPM and the larger nonfluorescent particles are silica. (B) The presence of DTAB allows for the nucleation and growth of TPM on silica. Fluorescent TPM is shown encapsulating non-fluorescent silica particles. Inset shows a close-up of an individual particle. See Supplementary Methods.

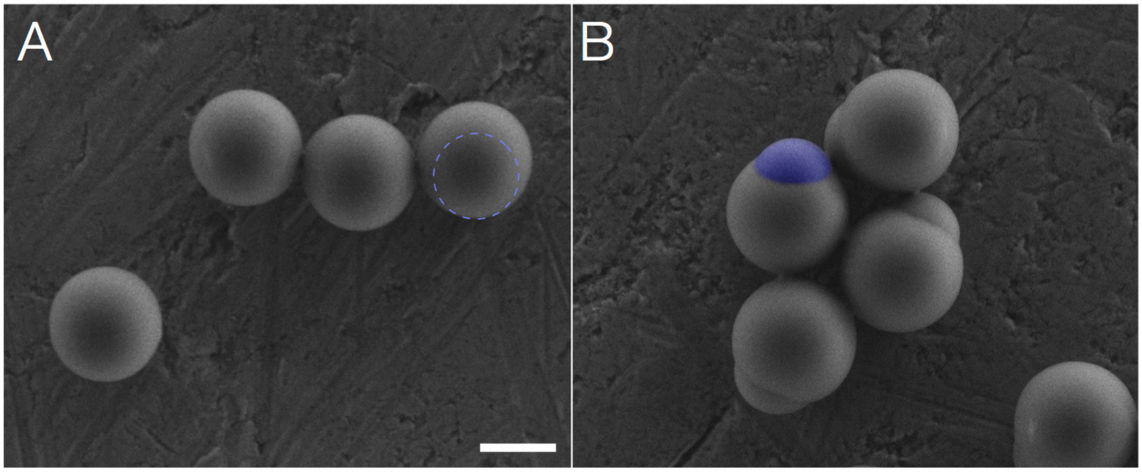

Supplementary Figure 9 (A) TPM oil added to a basic suspension of bare *p*-TPM particles results in the full encapsulation of the *p*-TPM particle in the oil phase. (B) Adding TPM oil to a basic suspension of *p*-TPM-*f108* results in the TPM oil wetting the polymerized particles with a finite contact angle (oil-phase polymerimerized prior SEM imaging). Scale bar is 1 μm. See Supplementary Methods.

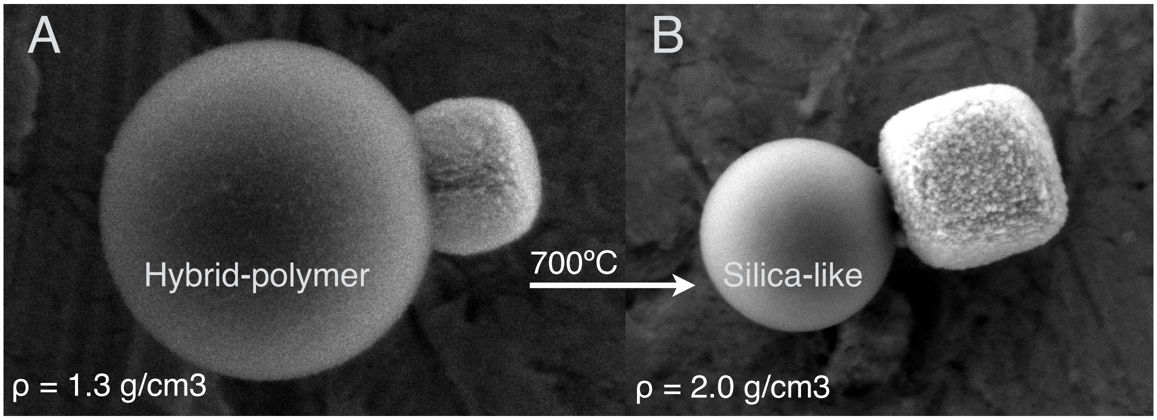

Supplementary Figure 10 (A) Polymerization of the TPM oil phase yields a TPM-hematite hybrid particle. (B) Calcination of the TPM-hematite hybrid particles yields hematite-silica hybrid particles.

Supplementary Methods

Homogeneous Nucleation of Oil Droplets - The homogeneous nucleation of 3-(Trimethoxysilyl)propyl methacrylate (TPM) results in monodispersed oil droplets with sizes that can typically range from 250 nm to 2.5 µm in diameter, depending on the experimental conditions. Since the oil precursor (TPM) is added to an aqueous solution containing ammonia, changing either of these conditions affects the droplet's size. Generally, a higher ammonia concentration results in smaller oil droplets while a higher TPM concentration results in larger oil droplets. This is demonstrated in Supplementary Fig.1, where TPM particles of different sizes are synthesized by tuning the ammonia and TPM concentrations. In a typical synthesis of TPM particles with a diameter of 1 µm, 1.5 mL of TPM oil is added to a solution containing 300 µL of NH3 (28% wt.) in 300 mL deionized water under stirring. Thanks to the acrylate moieties present in the TPM molecules, the oil droplets can be hardened via a simple radical polymerization. This hardening is achieved by adding about 0.5 mg of 2,2'-Azobis(2-methylpropionitrile) (AIBN) to a suspension of unpolymerized TPM oil droplets and heating the mixture at 80°C for 3 hours. Interestingly, we can also install a polymer brush on our TPM particles by introducing a block-copolymer. To do this, Pluronic® F-108 is added to the emulsions (after carrying out homogeneous nucleation as described above) to a final concentration of approximately 0.01% wt and then polymerizing the droplets using AIBN as previously stated. We use these features to prepare both solid particles with bare surfaces and particles with a grafted polymer brush (hereafter abbreviated *p*-TPM and *p*-TPM-*f108*,

respectively). These particles will later be used as seeds for heterogeneous nucleation studies.

Surface Charge Characterization - The charge of seed particles has been shown to have a drastic effect on the wetting of TPM on those seeds. For example, the full encapsulation of positive polystyrene occurs because of electrostatic attraction between the two materials, whereas TPM wets negatively charged polystyrene with a finite contact angle due to electrostatic repulsion. Since both hematite and bare *p*-TPM seed particles are fully encapsulated in the TPM oil phase during heterogeneous nucleation while TPM does not grow on silica seeds, we collected zeta potential data to compare the surface charge of the seed particles. Measurements were carried out on cubic hematite seeds, unpolymerized TPM oil droplets, and calcinated *p*-TPM particles using a Malvern Nano Series Zetasizer. Suspensions were prepared at a constant ionic strengh of 20 mM NaCl. Samples were left to equilibrate for 2 hours prior to each measurement. For all samples, the zeta potential was measured as a function of pH in the range from 2 to 12. The results are shown in Supplementary Fig. 2. The formation of a hydrophobe TPM layer occurs due to the condensation of silanols on the surface of particles containing hydroxyl groups during heterogeneous nucleation, as mentioned in the main text. Since heterogeneous nucleation is carried out at a pH of about 9, we compared the surface charge of hematite, unpolymerized TPM oil droplets, and calcinated *p*-TPM particles at this pH. While all three particles are negatively charged, silica carries an exceptionally high negative charge that we believe suppresses nucleation because of electrostatic repulsion between the surface and the precipitating silsesquioxane oligomers.

Formation of a TPM Hydrophobe Layer - Condensation of water soluble silanol-containing monomers results in the formation of large insoluble silsequioxane networks which have a proclivity for crashing out of the water phase and forming oil droplets. At first, this mechanism seems to be a concise explanation for both homogenous nucleation of TPM droplets, as well as heterogeneous nucleation of TPM on pre-existing seeds, until one considers the wetting angle of the TPM on seeds of varying material types. For example, TPM wets negatively charged polystyrene at a finite and measurable contact angle, while hematite is fully encapsulated in the oil phase. Though the two materials are negatively charged under our basic coating conditions, we see drastically differing morphologies between the two hybrid particles. To explain these observations, the condensation of TPM must occur on the surface of the seed particles, whereby the silanol moieties are chemically bound to the surface of preexisting seed particles. When comparing the surfaces of polystyrene and hematite, as well as the charged groups present on those surfaces (sulfonate and FeO-, respectively), we see that the sulfonate group on the polystyrene surface would not form a stable condensation product with the TPM precursor, whereas the Fe-O-Si bond is commonly found in many surface treatments involving silanol monomers. The formation of a hydrophobe layer on hematite is likely to occur in conditions where TPM is prone to condensing, such as the basic conditions we use to promote heterogeneous nucleation. We tested the presence of this hydrophobe layer by first coating hematite seeds with TPM and then washing the sample of all non-covalently linked TPM with ethanol, which TPM is soluble in.

First, the heterogeneous nucleation of TPM on hematite is carried out as previously described, whereby all oil droplets contain a single hematite seed inclusion. The suspension of hematite-containing oil droplets is then washed via two sedimentation and resuspension cycles in ethanol. After the second wash in ethanol, the particles are then resuspended in deionized water. At the same time, a control experiment was carried out where bare hematite particles were subject to identical washes. During ethanol washes, it is notable that both hydrophobized and bare hematite particles remain stable. After resuspension in water, there is a clear lack of stability in the hydrophobized hematite particles (as opposed to the control) that manifests as visible aggregates forming in deionized water (see Supplementary Fig. 3A).

We believe this instability is due to the new hydrophobic nature of the hematite afforded by the propyl methacrylate moiety of the hydrophobe TPM layer. The control experiment demonstrates that the ethanol washes do not have an inherent destabilizing effect, as bare hematite particles are subsequently stable in water (see Supplementary Fig. 3B). The native hematite surface can also be retrieved by etching off the hydrophobe layer in the same acidic etching conditions used to dewet the TPM droplets from hematite seeds. After 2 washes in 50 mM HCl, the seeds regain stability water.

As an independent check, we repeated the heterogeneous nucleation of TPM on hematite in the presence an adsorbing polymer. We use polyvinylpyrrolidone (PVP), as it is known to strongly adsorb onto hematite surfaces [1], since the adsorbed PVP should compete and interfere with chemical bonding of the silane oligomers to the substrate, thus altering the wetting of the hematite seed by the TPM oil. Indeed, under these conditions the TPM oil fails to fully wet the hematite seed particles. Instead, the TPM nucleates and grows on the hematite particles at a finite contact angle.

Selective Growth of TPM on Hematite - In Supplementary Fig. 5 we schematize how the process of oil-nucelation, dewetting, and polymerization can be iterated to grow multiple TPM lobes on seed particles. Polymerized dewetted hematite particles (such as those shown in Supplementary Fig. 6A and Supplementary Fig. 6D) can be further processed to install a second TPM lobe on the hematite peanut by repeating the oil nucleation step. The Janus-type seed particles contain two distinct surfaces, namely *p*-TPM-f108 and hematite. These materials have drastically different affinities for the incoming TPM, primarily due to accessibility of hydroxyl groups on the particle surface. This results in a single large droplet on the hematite surface, while the preexisting p-TPM-*f108* lobe is decorated with small physisorbed secondary TPM oil droplets that form via homogeneous nucleation (see Supplementary Fig. 5). This intermediate can then be treated with excess surfactant, such as F-108, which effectively dislodges the secondary TPM oil droplets from the *p*-TPM-*f108* lobe, most likely due to the emerging steric repulsion from the polymer shell. The final particle morphology shows growth of TPM on the hematite surface alone (Supplementary Fig. 6F).

Directing the Geometry of New Lobes - Since the dewetting of TPM from hematite is time dependent, it can be stopped at any point in time (via neutralization) to generate a range of morphologies with various levels of exposed hematite. When using peanut-

shaped hematite particles as seeds, quenching dewetting before the hematite particle is fully protruded results in two distinct hematite surfaces, separated by a thin TPM belt (see Supplementary Fig. 6A-B). Growing TPM on this seed results in the formation of two diametrically opposed lobes (see Supplementary Fig. 6C), in contrast to the singular lobe generated by a fully dewetted hematite seed particle (see Supplementary Fig. 6D-F).

Hematite Cube Synthesis - Though the synthetic method described by Sugimoto [2] for the synthesis of colloidal hematite cubes is rather straightforward, we initially obtained inconsistent results when attempting to synthesize these particles. In particular, polydispersed suspensions and oddly shaped particles (Supplementary Fig. 7A) were often produced instead of monodispersed micron-sized cubes (Supplementary Fig. 7B). The original procedure states that the aging of the hematite precursor (the ferric hydroxide gel) at 100°C takes about eight days, though we often observed that growth ceased after two or three days. On these occasions, the hematite particles that formed were always small and polydispersed. Such polydispersed particles form when there is not enough excess Fe^{3+} present after the formation of $Fe(OH)_3$ upon addition of NaOH to the $FeCl_3$ solution. The failed syntheses suggest that the initial $FeCl_3$ solution was less concentrated than the 2 M solution we thought we had prepared. Even previously unopened containers of $FeCl_3 \cdot 6H_2O$ powder were found to contain extra water content which accounted for a large percentage of the weight of the powder, due to the hygroscopic nature of $FeCl_3 \cdot 6H_2O$. However, after taking the extra moisture present in $FeCl_3 \cdot 6H_2O$ into account (using thermo- gravimetric analysis for example) the synthesis can be carried out simply by adjusting either the amount of $FeCl_3 \cdot 6H_2O$ used or NaOH added.

Oil Nucleation on Silica Seeds - Attempting to nucleate TPM onto Stober silica (following the procedure described in the main text for hematite) results solely in the homogeneous nucleation of separate TPM oil droplets, which leads to a bidispersed suspension (Supplementary Fig. 8A). We speculate that this low wettability is caused by the particularly strong negative surface charge of silica. This hypothesis seems to be supported by the fact that the addition of small amounts of a cationic surfactant such as dodecyl trimethyl ammonium bromide (DTAB) is sufficient to promote the heterogeneous nucleation of the oil on the silica seeds and to yield fully encapsulated seeds (Supplementary Fig. 8B).

Bare and Polymer-Coated Seeds - To further investigate the mechanism of heterogeneous nucleation of the oil phase on colloidal substrates, we carried out nucleation experiments on both bare and polymer-grafted colloidal surfaces. We observed that bare *p*-TPM seeds are fully wetted by the oil (Supplementary Fig. 9A), while TPM oil wets *p*-TPM-*f108* seeds with a finite contact angle (Supplementary Fig. 9B). This can be explained by considering that the presence of a polymer brush (here Pluronic® F-108) can interfere with the mechanism of the formation of a hydrophobe monolayer layer described in the main text. In fact, while the hydroxyl groups on the bare particles are free to undergo condensation reactions with the silanols generated in situ, the hydroxyl groups on the *p*-TPM-*f108* particles are less accessible due to steric considerations brought about by the surfactant.

Minimizing Secondary Nucleation - The heterogeneous nucleation of oil droplets is generally achieved by simply adding the TPM oil precursor to a seed suspension in the presence of ammonia. However, this method often causes the formation of secondary nano-sized droplets via homogeneous nucleation that require separation. To minimize secondary nucleation, a solution of pre-hydrolyzed TPM can be added drop-wise to the basic seed suspension. This slow addition allows us to maintain the concentration of the reactive oil precursor at low levels, preventing the homogeneous nucleation of TPM oil droplets. In our experiments, the solution of pre-hydrolyzed TPM is prepared by adding the precursur to water in a volume ratio of 1 to 10 and then vigorously stirring this mixture until a clear and homogeneous solution is obtained. The hydrolyzed product can then be dripped at a rate of one drop every two seconds into the same basic suspension of seed particles while stirring gently. The addition of hydrolyzed TPM continues until the desired size is reached and progress can be monitored via optical microscopy.

Dewetting Experiments

As we described in the main text, the dewetting of hematite seeds can be triggered by a chemical treatment or by light.

Acid-Triggered Dewetting - Under acidic conditions, unpolymerized TPM oil droplets carry a very low surface charge (Supplementary Fig. 2), which can result in a catastrophic coalescence. To increase the colloidal stability of the TPM oil droplets at a low pH, Pluronic® F-108 is added to the system to a final concentration of about 0.01% wt. The time required for dewetting to occur varies depending on HCl concentration, temperature, and the age of the oil droplet. In particular, high temperatures and high HCl concentrations both result in an increase of the hematite etching rate, which reduces the time for dewetting to be observed. We also observed that older oil droplets generally require longer periods of time for dewetting to be observed. This aging effect may be caused by a gradual increase of the silsesquioxanes molecular weight which results in a time-dependent oil viscosity. In our experiments, we were able to induce dewetting for concentrations as low as 20 mM HCl, with no upper limit, except for the fact that hematite is etched very rapidly at high concentrations of HCl. After dewetting has occurred, the TPM-hematite hybrid droplets are isolated from the secondary nucleation via a gentle sedimentation followed by resuspension in deionized water. Washing is repeated until a neutral pH is reached. The TPM oil phase is polymerized using AIBN as previously described.

Base-Triggered Dewetting - When the TPM-hematite hybrid particles are suspended in 60 mM NaOH at 65°C, dewetting is typically observed in 1.5 to 2 hours, depending on the age of particles. Prolonged suspension in basic conditions results in the detachment of the TPM oil droplets from the hematite seed particles. In contrast to acid-triggered dewetting, etching with NaOH does not require the addition of a surfactant to the emulsion in order to keep the emulsion colloidally stable, since at a high pH both hematite and TPM oil are highly charged (Supplementary Fig. 2). The dewetting process can be monitored via optical microscopy, and various intermediate stages can be fixed by

neutralizing the NaOH with HCl and polymerizing the oil phase with AIBN as previously described.

Light-Triggered Dewetting - Dewetting of the oil phase from hematite seeds can also be triggered by light. Typically, the TPM-hematite hybrid particles are suspended in 2.5% wt. H_2O_2. Dewetting is then triggered by exposing the TPM-hematite hybrid particles to green or blue light. The extent of dewetting of TPM from the seeds can be controlled by varying the time that the particles are exposed to the light. Additionally, dewetting proceeds faster in particles suspended in higher concentrations of H_2O_2. Prolonged exposure to light results in the complete detachment of the oil droplet from the hematite seeds.

Silica - Dewetting of TPM from silica seeds has been achieved using NaOH under the same conditions used for hematite, with the exception that heating is not required because dewetting proceeds sufficiently fast at room temperature. In contrast to hematite seeds, which are capable of being dewetted under acidic conditions and with light, TPM has only been dewetted from silica under basic conditions. Since hematite has been shown to etch in acidic conditions, whereas silica and unpolymerized TPM oil droplets are less susceptible to acid etching, the lack of dewetting at low pH is an expected result.

Titania - Like silica, oil can be dewetted from titania seed particles, though at harsher conditions, requiring 90 mM NaOH and up to 3 hours for dewetting to occur at 65°C.

Supplementary References

[1] Sacanna, S.; Rossi, L.; Kuipers, B.; Philipse, A. *Fluorescent monodisperse silica ellipsoids for optical rotational diffusion studies*, Langmuir, **22**, 1822–1827, (2006).

[2] Sugimoto, T.; Wang, Y.; Itoh, H.; Muramatsu, A. Colloids And Surfaces A-Physicochemical And Engineering Aspects, *Systematic control of size, shape and internal structure of monodisperse alpha-Fe_2O_3 particles*, **134**, 265–279, (1998).
